# Multimodal imaging of retinal findings in syndactyly, telecanthus, anogenital, and renal malformations (STAR) syndrome

**DOI:** 10.1016/j.ajoc.2022.101284

**Published:** 2022-01-20

**Authors:** Alexa L. Li, Shyamanga Borooah, Eric Nudleman

**Affiliations:** Shiley Eye Institute, Department of Ophthalmology, University of California at San Diego, La Jolla, CA, USA

**Keywords:** Syndactyly, Telecanthus, Anogenital and renal malformations, Retina

## Abstract

**Purpose:**

To report multimodal imaging of novel retinal findings in a case of syndactyly, telecanthus, anogenital, and renal malformations (STAR) syndrome.

**Observations:**

A 5-year old patient with STAR syndrome, an ultra-rare developmental disorder composed of syndactyly, telecanthus, anogenital, and renal malformations, was found to have bilateral macular yellow pigmentary changes and peripheral retinal pigment epithelial changes in a radial pattern highlighted by fundus autofluorescence (FAF) imaging. Optical coherence tomography (OCT) of the macula revealed foveal hypoplasia, ellipsoid zone disruption, and outer retinal atrophy suggestive of a retinal degeneration. OCT angiography found no significant abnormalities, and oral fluorescein angiography revealed staining in areas of atrophy in both eyes.

**Conclusions and Importance:**

This case displays the first report of multimodal imaging of retinal manifestations in STAR syndrome, revealing bilateral foveal hypoplasia, outer retinal macular atrophy, and peripheral retinal pigment epithelial changes. Further studies and long-term follow-up are warranted to determine if patients with STAR syndrome have an underlying progressive retinal degeneration.

## 1 Introduction

STAR syndrome is an ultra-rare X-linked dominant disorder originally coined by Unger et al. as an acronym describing the characteristic phenotype of this syndrome: syndactyly, telecanthus, anogenital, and renal malformations.^1^ This developmental disorder, associated with male lethality, results from mutations in the *family with sequence similarity 58, member A* (*FAM58A*) gene on chromosome Xq28.^1^ The *FAM58A* gene encodes the protein Cyclin M, which interacts with cyclin-dependent kinase 10 (CDK10). CDK10 plays an important role in cell division and proliferation.^2^^,^^3^ To our knowledge, there are only 15 patients reported with STAR syndrome in the literature.^1^^,^^3^, ^4^, ^5^, ^6^, ^7^, ^8^, ^9^

STAR syndrome can be associated with ocular abnormalities. Ocular manifestations include telecanthus and eyelid anomalies, in addition to peripheral anterior synechiae in the anterior segment.^8^ Retinal findings have also been described, including macular drusen^8^ and macular hypoplasia.^1^ In this case report, we present findings of bilateral macular outer retinal atrophy, foveal hypoplasia, and peripheral retinal pigment epithelial changes in a patient with STAR syndrome. This is the first report using multimodal imaging to document the retinal changes in this ultra-rare developmental syndrome.

## 2 Case report

This female patient was born at term (37 weeks and 5 days gestational age) with a birth weight of 2550 g by Cesarean section for breech presentation after a pregnancy complicated by oligohydramnios. Family history was unremarkable and parents denied consanguinity. At birth, the patient was noted to have multiple congenital abnormalities, including syndactyly of the right and left 3rd, 4th, and 5th toes, telecanthus, anteriorly displaced and imperforate anus, bilateral hydronephrosis, ano-vaginal fistula, tethered spinal cord, left sensorineural hearing loss, clitoromegaly, accessory nipple, and bilateral club feet. Additional diagnostic studies revealed a ventricular septal defect and patent ductus arteriosus which closed without intervention. She was also found to have hydrocephalus but did not require a shunt placement.

The patient underwent the following surgical procedures: release of tethered spinal cord at 3 months, repair of ano-vaginal fistula at 4 months, laparoscopic Ladd's procedure with appendectomy for intestinal malrotation noted at 6 months, and ileostomy and multiple surgical revisions for neurogenic bladder and bowel at 4 years of age.

An ophthalmological examination was performed at an outside pediatric ophthalmology service at age 7 months; that exam noted mild telecanthus and pigment mottling in the macula of both eyes. She was examined again at 1 year and 2 years of age and was observed to have similar bilateral pigmentary changes in the macula.

The patient then presented at our institution at 5 years of age for ophthalmological examination. At this visit, visual acuity was 20/200 in the right eye and 20/160 in the left eye with correction. Cycloplegic refraction was −3.50 + 4.50 × 095 in the right eye and −3.25 + 3.75 × 080 in the left eye. External examination revealed mild telecanthus, and anterior segment examination was normal in both eyes. Intraocular pressure was 14 mmHg in the right eye and 15 mmHg in the left eye.

**Fig. 1:**
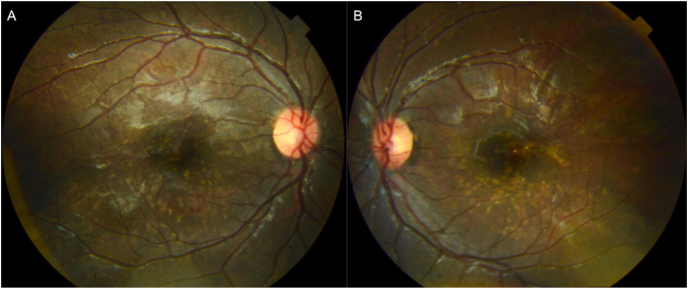
Color fundus photos of the right (A) and left (B) eyes reveal yellow drusen-like pigmentary changes in the macula of both eyes. (For interpretation of the references to colour in this figure legend, the reader is referred to the Web version of this article.)

**Fig. 2:**
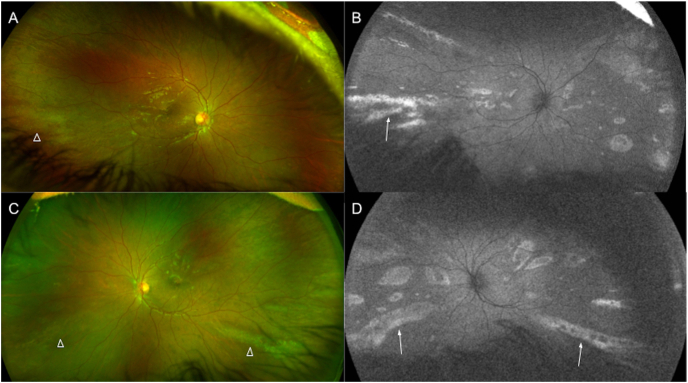
Color Optos photos of the right (A) and left (C) eyes reveal yellow pigmentary changes in the macula and peripheral retinal pigment epithelial changes (arrowheads) in both eyes. Fundus autofluorescence (B and D) highlights a radial pattern of hyperautofluorescent changes (arrows) in the periphery of both eyes. (For interpretation of the references to colour in this figure legend, the reader is referred to the Web version of this article.)

**Fig. 3:**
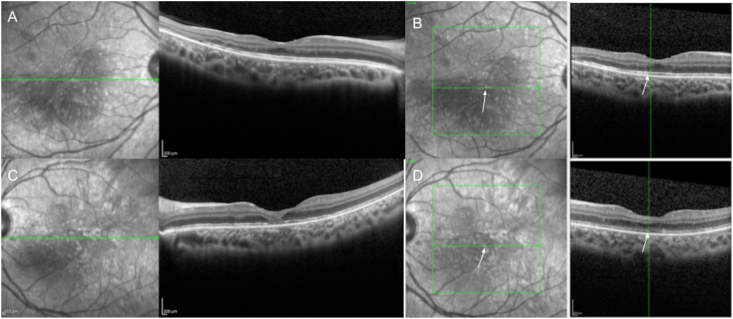
Optical coherence tomography (OCT) of the macula demonstrates outer retinal atrophy and disruption in the ellipsoid zone (EZ) in both eyes. In the right eye (A), grade 1 foveal hypoplasia was present, while there was atypical foveal dysplasia in the left eye (C) with disruption of the inner segment ellipsoid band. Co-localization of the drusen-like deposits (arrows) on near-infrared reflectance image with OCT did not reveal any visible deposits of drusen between the RPE and Bruch's membrane in the right (B) or left (D) eyes.

**Fig. 4:**
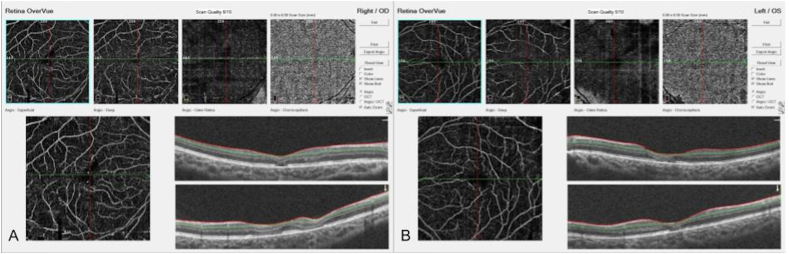
OCT Angiography shows normal vessel density and a normal foveal avascular zone in the right (A) and left (B) eyes.

**Fig. 5:**
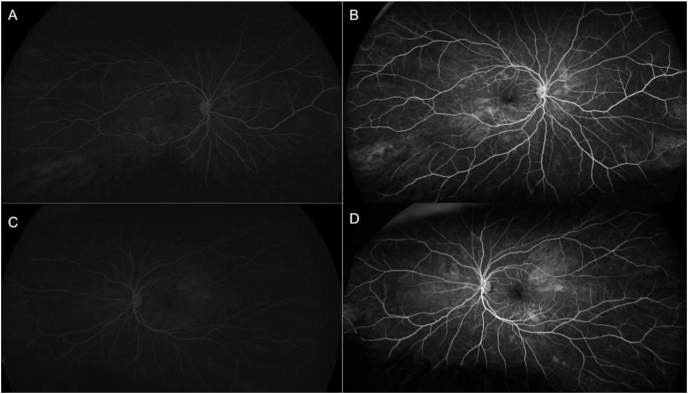
Oral fluorescein angiogram (FA) in the right eye at 6 minutes and 2 seconds (A) and at 11 minutes and 33 seconds (B) reveal staining in areas of atrophy. FA in the left eye at 5 minutes and 40 seconds (C) and at 16 minutes and 6 seconds demonstrate a similar pattern of patchy staining in areas of atrophy.

On posterior segment examination, the optic nerves were normal without evidence of optic nerve hypoplasia. Retinal examination was remarkable for yellow drusen-like deposits in the macula (Fig. 1) and peripheral retinal pigment epithelial (RPE) changes in both eyes (Fig. 2). These peripheral changes were highlighted on fundus autofluorescence (FAF) as patchy and linear hyperautofluorescent changes in a radial pattern (Fig. 2). Optical coherence tomography (OCT) of the macula revealed outer retinal atrophy and disruption of the ellipsoid zone (EZ) (Fig. 3). There was also increased hyperautofluorescence in the areas of EZ disruption and outer retinal atrophy, suggesting that loss of retina led to exposure of more RPE and thus increased hyperautofluorescence. Interestingly, co-localization of the drusen-like deposits on near-infrared reflectance imaging (NIR) with OCT did not reveal any discrete deposits at the level of the RPE or Bruch's membrane (Fig. 3). There was persistence of inner retinal layers at the fovea suggestive of foveal hypoplasia. In the right eye, grade 1 foveal hypoplasia was present with a nearly normal foveal pit.^10^ There was atypical foveal dysplasia in the left eye with disruption of the inner segment ellipsoid band. OCT angiography did not reveal a choroidal neovascular membrane and showed normal vessel density and normal foveal avascular zone in both eyes (Fig. 4). Oral fluorescein angiography exhibited staining in areas of atrophy in both eyes (Fig. 5).

Microarray analysis revealed a likely pathogenic 150 kilobase deletion of chromosome Xq28 (arr[hg19] Xq28(152,834,159–152,984,574)x1) containing 6 genes including *FAM58A*, which established the diagnosis of STAR syndrome. Besides *FAM58A*, none of the other genes were associated with any previously reported ophthalmic manifestations. The parents also underwent genetic testing and were not found to have the same mutation.

## 3 Discussion

A phenotypic spectrum of ophthalmic manifestations has been described in patients with STAR syndrome. Telecanthus was defined by Unger et al. as a cardinal characteristic feature of this syndrome.^1^ Additional eyelid findings include lower eyelid coloboma,^1^ congenital ptosis,^5^ and bilateral medial upper eyelid prominences with madarosis, as reported for the first time by Orge et al.^8^ in a mother-daughter pair. Strabismus can occur in this syndrome due to bilateral Duane's anomaly^3^ and accommodative esotropia.^5^ In the anterior segment, peripheral anterior synechiae without evidence of coexisting glaucoma was observed in one of the patients at 6 months of age, suggestive of a possible structural abnormality as seen in anterior segment dysgenesis disorders.^8^ Both hyperopic^5^ and myopic^1^ refractive states have been reported. Our patient presented with mild telecanthus noted at birth and but otherwise did not have other anterior segment abnormalities.

In the posterior segment, reported optic nerve anomalies of STAR syndrome include peripapillary atrophy and optic nerve hypoplasia.^8^ Retinal findings have also been previously described in four patients with this syndrome. Unger et al. reported a patient with −5 diopters of myopia and a dystrophic retina in one patient in his case series.^1^ In the macula specifically, macular hypoplasia^1^ and soft macular drusen^8^ have been described in three patients. It is uncertain if the underlying retinal findings were similar in these reports, as not all cases had fundus photography.

In our patient, the primary ocular manifestation was retinal in the form of macular yellow pigmentary changes and peripheral, radial RPE changes. Interestingly in our patient, the macular findings appeared similar to those depicted in the fundus photos in Orge's case series of a mother-daughter pair, which the authors described as “soft macular drusen”.^8^ Yellow drusen-like deposits were similarly observed in the macula of both eyes of our patient. However, co-localization of the near-infrared reflectance image with OCT did not reveal any visible deposits of drusen between the RPE and Bruch's membrane. Instead, there was ellipsoid zone disruption and outer retinal atrophy, suggestive of early onset degeneration of the outer retina. OCT imaging also revealed foveal hypoplasia with absence of extrusion of the plexiform layers in the right eye. In particular, in the left eye, there was also an associated disruption in the ellipsoid zone in the fovea, indicative of atypical foveal dysplasia.^10^ Interestingly, the radial pattern of the peripheral retinal pigment epithelial changes highlighted on fundus autofluorescence imaging seen in our patient has also been observed in other X-linked inherited retinal disorders such as retinitis pigmentosa, hypothesized to be in part due to X-chromosome inactivation and mosaicism.^11^

The underlying pathogenesis in this rare genetic disorder is currently not well-understood but may be related to regulation of the cell cycle.^1^ Mutations in the X-linked *FAM58A* gene encoding Cyclin M (CycM) lead to a deficiency in the formation of the CDK10/CycM protein kinase, which plays a key role in the regulation of ciliogenesis.^2^ Loss of the *FAM58A* gene causes haploinsufficiency, leading to intrauterine mortality in males. A primary ciliopathy may be the mechanism by which STAR syndrome causes retinal, renal, anogenital, and digital anomalies. Guen and colleagues discovered abnormal, elongated cilia and dilated renal tubules in a kidney biopsy of a STAR syndrome patient, suggesting the presence of a ciliogenesis defect.^2^ Furthermore, Unger et al. hypothesized that a proliferation defect and degeneration of photoreceptors can occur in this syndrome,^1^ as retinal abnormalities were observed in homozygous *cyclin D1* knockout mice. A limitation of our report includes the possibility that the retinal findings were related to a contiguous gene syndrome, as the deletion of Xq28 (arr[hg19] Xq28(152,834,159–152,984,574)x1) contained regions neighboring *FAM58A*.

## 4 Conclusions

This case report presents the first documented multimodal imaging findings of retinal manifestations in STAR syndrome. OCT imaging revealed foveal hypoplasia as well as outer retinal atrophy and ellipsoid zone disruption suggestive of a retinal degeneration. Fundus autofluorescence imaging can highlight a characteristic, radial pattern of hyperautofluorescence as seen in other X-linked inherited retinal disorders. Questions remain as to whether the retinal changes are progressive, and further studies and long-term ophthalmic follow-up of patients with STAR syndrome are warranted.

## Patient consent

The patient's legal guardian consented to publication of the case in writing.

## Authorship

All authors attest that they meet the current ICMJE criteria for Authorship.

## Funding

No funding was received for this work.

## Intellectual property

We confirm that we have given due consideration to the protection of intellectual property associated with this work and that there are no impediments to publication, including the timing of publication, with respect to intellectual property. In so doing we confirm that we have followed the regulations of our institutions concerning intellectual property.

## Research ethics

We further confirm that any aspect of the work covered in this manuscript that has involved human patients has been conducted with the ethical approval of all relevant bodies and that such approvals are acknowledged within the manuscript.

IRB approval was obtained (required for studies and series of 3 or more cases).

Written consent to publish potentially identifying information, such as details or the case and photographs, was obtained from the patient(s) or their legal guardian(s).

## Declaration of competing interest

No conflict of interest exists.
